# Changes in Foliar Water Uptake Caused by Water Stress in *Cattleya* Lindl. (Orchidaceae)

**DOI:** 10.1111/ppl.71047

**Published:** 2026-09-02

**Authors:** Jéssica Ferreira de Lima, Gisely de Souza Santos, Denis Coelho de Oliveira, Ana Silvia Franco Pinheiro Moreira

**Affiliations:** ^1^ Instituto de Biologia, Laboratório de Fisiologia Vegetal Universidade Federal de Uberlândia (UFU) Uberlândia Minas Gerais Brazil; ^2^ Instituto de Biologia, Laboratório de Anatomia, Desenvolvimento Vegetal e Interações Universidade Federal de Uberlândia (UFU) Uberlândia Minas Gerais Brazil

**Keywords:** adaptive strategies, apoplastic pathway, epiphytes, organic acids, water status

## Abstract

In response to water restrictions imposed by the epiphytic environment, orchids have developed strategies to increase water‐use efficiency, such as the expression of Crassulacean Acid Metabolism (CAM) and the foliar water uptake (FWU). In a climate change scenario, FWU may emerge as a strategy to reduce water stress during drought. In this study, we propose that, in *Cattleya forbesii* Lindl. and *Cattleya guttata* Lindl., drought alters the leaf water balance, increases CAM expression, and may consequently increase the FWU rate. To test this hypothesis, drought stress was induced by restricting water for 30 days in *C. forbesii* (*n* = 4) and *C. guttata* (*n* = 6). After that, we evaluated the relative water content (RWC), overnight organic acid accumulation (ΔH^+^), and FWU capacity. To verify the pathway of water entry into the leaves, we used the apoplastic marker Lucifer Yellow. We observed that in both *Cattleya* species, drought impacted leaf water status, photosynthetic activity, and FWU capacity. After water restriction, the leaf water status decreased, impairing photosynthetic activity in both species (lower RWC, potential quantum yield of PSII, and quantum efficiency under light‐adapted conditions), whereas nonphotochemical quenching increased. Despite minor changes in ΔH^+^ values, FWU increased following drought, partially supporting our hypothesis. In both *Cattleya* species, LY solution diffused through the epidermis and reached the mesophyll; however, in *C. guttata,* this occurred only through the abaxial epidermis. These results demonstrate that FWU may serve as a strategy for *Cattleya* species to cope with water scarcity intensified by drought periods.

## 1 Introduction

The epiphytic environment imposes a series of restrictions on plants, including greater fluctuations in water availability, nutrient availability, and light incidence (Benzing 1990). One of the most concerning factors is water scarcity, since the hydration of epiphytes depends exclusively on atmospheric sources (Benzing 1990; Zotz et al. 2010; Zotz 2016; Zhang et al. 2016; Rosado‐Calderón et al. 2020). Orchids are found in various types of habitats, but approximately 75% of the species are epiphytes (Zotz et al. 2021). The success of this group in the tree canopy is due to the morphological and physiological strategies of their vegetative organs, which favor greater water use efficiency (Benzing 1990; Zotz and Hietz 2001; Zhang et al. 2018). Succulent leaves and the expression of Crassulacean Acid Metabolism (CAM) are examples of these strategies (Gibson 1982; Cushman 2001; Zhang et al. 2015; Chukina et al. 2024). The succulence of the leaves increases water storage in the tissue and functions as a form of escape from water stress (Ogburn and Edwards 2012). Moreover, it has a direct relationship with CAM metabolism, since the organic acids produced at night can be stored in the vacuoles of mesophyll cells (Cushman 2001; Moreira et al. 2009, 2013).

The CAM pathway is a photosynthetic process whose water use efficiency is greater than that of other photosynthetic pathways (C3 or C4), since atmospheric carbon dioxide (CO_2_) fixation occurs at night, when water loss through transpiration is minimal (Winter et al. 2005; Freschi et al. 2007; Castillo et al. 2016). In general, CAM plants separate CO_2_ capture and fixation by the Calvin–Benson cycle in time (Osmond 1978; Ting 1985). During the night, the stomata open, and atmospheric CO_2_ is fixed via phosphoenolpyruvate carboxylase (PEPcase) and stored in vacuoles, usually in the form of organic acids (mainly malate). In the morning, malate is decarboxylated by the NADP‐malic enzyme, and the released CO_2_ is supplied to the Calvin–Benson cycle through the carboxylase activity of ribulose 1,5‐bisphosphate carboxylase oxidase (RuBisCO), where it is incorporated into organic compounds (Cushman 2001).

Water scarcity can affect the water status and photosynthetic efficiency of epiphytes, often assessed by the reduction in relative leaf water content (RWC; Anjum et al. 2011) and by changes in parameters related to chlorophyll *a* fluorescence (Stancato et al. 2001; de la Rosa‐Manzano et al. 2015, 2017; Tay et al. 2015; Sharma et al. 2020). For example, six species of epiphytic orchids under water stress conditions were previously studied by Tay et al. (2015), who reported that changes in RWC were accompanied by a decrease in photosystem II (PSII) quantum efficiency. Photochemical performance in response to water deficit has been analyzed through chlorophyll *a* fluorescence, with studies addressing light‐adapted PSII quantum efficiency (ϕ_PSII_), PSII potential quantum yield (*F*_v_/*F*_m_), nonphotochemical quenching (NPQ), and the fluorescence decay rate (*R*_fd_; Genty et al. 1989; Horton and Ruban 1992; Lichtenthaler and Miehe 1997; Stancato et al. 2001; Baker 2008; Oxborough 2004; Masrahi et al. 2015; Ceusters et al. 2019). The light‐adapted PSII quantum efficiency (ϕ_PSII_) and *F*_v_/*F*_m_ indicate the photochemical efficiency in light and the degree of damage to the PSII reaction center, respectively (Kitajima and Butler 1975; Björkman and Demmig 1987; Maxwell and Johnson 2000). Nonphotochemical quenching functions as a photoprotective mechanism, preventing damage to the photosynthetic apparatus through the thermal dissipation of excess light energy absorbed by PSII (Nobel 2009; Takahashi and Badger 2011; Demmig‐Adams et al. 2006, 2012). The fluorescence decline ratio, also known as the vitality index, is related to CO_2_ assimilation, and a decrease in this value may indicate damage to the photosynthetic apparatus (Babani and Lichtenthaler 1996; Lichtenthaler et al. 2005; Rao et al. 2021).

A complementary process that helps vascular epiphytes to better cope with water deficits is foliar water uptake (FWU; Gotsch et al. 2015; Pan et al. 2021). FWU has already been demonstrated in several species distributed across various ecosystems (Limm et al. 2009; Eller et al. 2013, 2016; Goldsmith 2013; Steppe et al. 2018; Bryant et al. 2021), including epiphytic orchids (Pan et al. 2021; Lima et al. 2023). Foliar water uptake occurs in response to a water potential gradient caused by the difference in vapor pressure between the atmosphere and the interior of the leaf, causing water to pass through the leaf surface and promote hydration of leaf tissues (Burgess and Dawson 2004). Water enters leaves through different pathways, including the cuticle (Ritpitakphong et al. 2016; Fernández et al. 2017; Gui et al. 2021), trichomes (Schreel et al. 2020; Gui et al. 2021), hydathodes (Martin and von Willert 2000; Boanares et al. 2019; Fradera‐Soler et al. 2024), and stomata (Berry et al. 2014; Binks et al. 2020; Guzmán‐Delgado et al. 2021). Most of the pathways used for FWU are still unknown, as they vary from species to species (Eller et al. 2016; Schreel and Steppe 2020; Liu et al. 2021; Gui et al. 2021). However, FWU has many advantages in terms of leaf physiology, as it increases hydration (water potential), reduces stomatal conductance, increases water use efficiency, and consequently, can influence the photosynthetic capacity of plants in different ways (Limm et al. 2009; Eller et al. 2016; Vesala et al. 2017; Berry et al. 2019).

In a climate change scenario in which drought events will be more frequent and prolonged (Schreel and Steppe 2020), FWU appears to be an interesting strategy for alleviating water stress, especially for plants that suffer from this condition daily, such as epiphytes. Given that epiphytic orchids capable of expressing CAM metabolism can absorb water through their leaves (Lima et al. 2023), and that FWU is influenced by leaf water balance, we experimentally tested whether water deficit reduces leaf water content, thereby increasing the water potential gradient between the leaf and the external environmental and favoring water entry. In addition, we evaluated whether increased CAM expression, associated with nocturnal accumulation of organic acids, could further contribute to increase FWU rates. We used two epiphytes capable of expressing CAM metabolism, *Cattleya forbesii* and *Cattleya guttata*, as study models. We hypothesize that both species, after being subjected to water deficit conditions for 30 days, will show a reduction in leaf water status and photosynthetic activity, while their leaf water absorption capacity (FWU) will be maximized. We also expect an increase in CAM expression (i.e., higher accumulation of organic acids) under drought conditions. Thus, we proposed to: (1) evaluate changes in the water status and photosynthetic activity of the leaves of *C. forbesii* and *C. guttata* subjected to drought conditions; (2) test whether FWU rates increase after drought; and (3) investigate the apoplastic pathway of water uptake in both species.

## 2 Materials and Methods

### 2.1 Plant Species and Experimental Design

*C. forbesii* Lindl. and *C. guttata* Lindl. are epiphytic species endemic to Brazil, found in the Atlantic Forest (van den Berg 2020), and express CAM metabolism (Lima et al. 2023). Orchids are widely used as ornamental plants and have high commercial value; among the cultivated genera, *Cattleya* is very popular (Teixeira Da Silva et al. 2014; Colombo et al. 2017). According to the Ministry of the Environment (Ordinance No. 443, issued on December 17, 2004), several Brazilian orchids are at risk of extinction, including *C. guttata* . Adult samples of both species were available at the Institute of Biology's greenhouse on the Umuarama campus of the Federal University of Uberlândia (UFU), Uberlândia, Minas Gerais. The greenhouse is maintained under natural temperature and humidity conditions, with the region's climate characterized by a dry winter (April–September) and a rainy summer from October–March, as classified by the Köppen system (Alvares et al. 2013). In the year prior to the experiment (May 2022–April 2023), the annual average temperature was 24.38°C, with the maximum temperature recorded being 35.4°C and the minimum temperature being 7.9°C (INMET).

**FIGURE 1:**
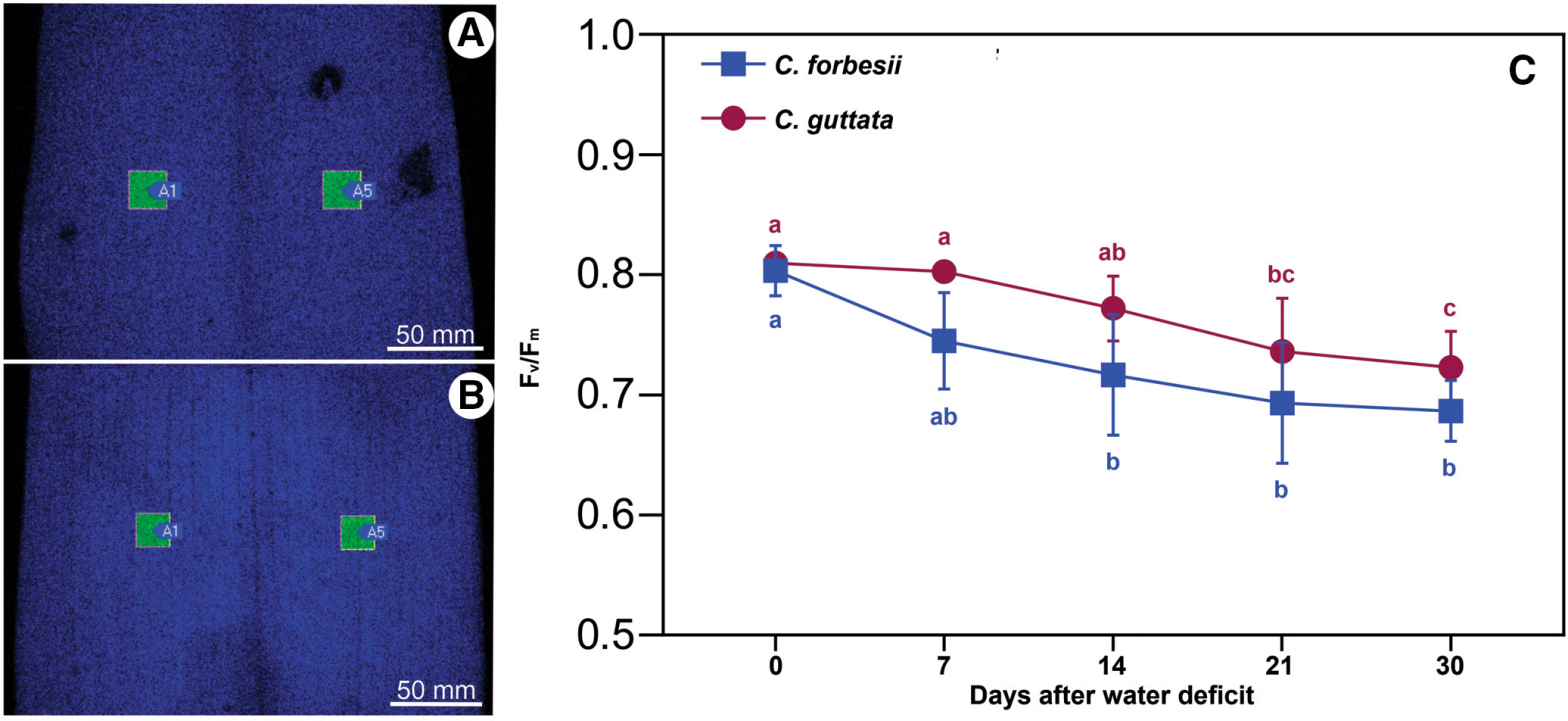
Evaluation of chlorophyll *a* fluorescence in the leaves of *Cattleya forbesii* (A) and *C. guttata* (B) subjected to drought. The analyses were conducted weekly until a significant decrease in the potential quantum yield of PSII (F_v_/F_m_) was observed (C). The analyses used the *quenching* protocol available in the FluorCam 7.0 software (Handy FluorCam, PSI). Lowercase letters represent significant differences in *F*_v_/*F*_m_ between the times (0, 7, 14, 21, and 30 days) analyzed in the leaves of *C. forbesii* (*χ* ^2^ = 40.11, Df = 4, *p* < 0.0001, *R*_*m*_^2^ = 0.65 and *R*_*c*_^2^ = 0.77; lowercase letters in purple) and *C. guttata* leaves (*χ* ^2^ = 51.43, Df = 4, *p* < 0.0001, *R*_*m*_^2^ = 0.68 and *R*_*c*_^2^ = 0.68; lowercase letters in purple) via Mixed Linear Models (LMMs; *p* < 0.05), and we compared the means using Tukey's adjusted paired comparisons (“emmeans” package; *p* < 0.05).

In May 2023, seven individuals of *C. forbesii* and 11 of *C. guttata* underwent acclimatization in the greenhouse for 1 year and were irrigated with water three times a week during the dry season. The experiment was subsequently conducted at the Laboratório de Anatomia, Desenvolvimento Vegetal e Interações (LADEVI) of the Federal University of Uberlândia, in a growth room under controlled light (29.27 mol m^−2^ s^−1^) and temperature conditions (24°C). Some individuals were kept as controls, and others were subjected to drought (treatment). For the control individuals of *C. forbesii* (*n* = 3) and *C. guttata* (*n* = 5), irrigation was maintained three times a week. To induce drought (treatment), irrigation of *C. forbesii* (*n* = 4) and *C. guttata* (*n* = 6) individuals was suspended until signs of water stress were detected. In this way, the onset of water stress was determined by a decrease in the potential quantum yield (*F*_v_/*F*_m_); photosynthetic performance was assessed weekly by measuring chlorophyll *a* fluorescence with an imaging fluorometer (Handy FluorCam PSI, Czech Republic). The experiment was concluded after 30 days, when the plants exhibited signs of significant water stress, as indicated by a decrease in the potential quantum yield (*F*_v_/*F*_m_) values compared to those in the control group (Figure 1).

### 2.2 Evaluation of Structural Parameters and Leaf Water Balance

Fragments from the middle region of the leaves of *C. forbesii* and *C. guttata* were collected for anatomical description, fixed in FAA_70_ (formaldehyde, acetic acid, 70% ethyl alcohol, 1:1:18 v/v) for 48 h, and then stored in 70% ethanol (Johansen 1940). To prepare the histological slides, transverse and longitudinal sections were prepared manually using razor blades. The fragments were clarified in 50% sodium hypochlorite, washed in distilled water, and then stained with 0.5% Alcian Blue and 0.5% safranin (9:1 v/v; Kraus and Arduin 1997). The slides were mounted with 50% glycerin and photographed with an ICC50 HD digital camera attached to a DM500 microscope (Leica).

To determine relative water content (RWC), water saturation content (SWC), and leaf mass per area (LMA), 1 cm^2^ leaf fragments were collected from each individual of *C. forbesii* and *C. guttata* , including both control plants and those subjected to 30 days of drought. The samples were collected between 7 and 8 h. The RWC was used to assess the water status of the leaves, calculated using the equation RWC = [FM—DM]/[TM—DM] * 100, where FM is the leaf fresh mass, DM is the dry mass after drying the material in an oven at 50°C until it reaches a constant weight, and TM is the turgid mass after hydration for 24 h (Turner 1981). As an indicator of leaf succulence, we used the water saturation content (SWC), which was determined by the formula *SWC =* (*TM—DM*)/*DM* (Ogburn and Edwards 2012). Leaf mass per area (LMA) was used to assess tissue density, obtained by the formula *LMA = DM/A*, where A corresponds to the fragment area, according to Witkowski and Lamont (1991).

### 2.3 Chlorophyll a Fluorescence

To obtain the fluorescence parameters of chlorophyll *a* after 30 days of experimentation, an image‐based fluorescence meter (Handy FluorCam, Photo Systems Instrument, Czech Republic) was used to calculate the Kautsky effect according to the Quenching protocol (FluorCam 7.0 software, Photo Systems Instrument), with saturating light at 1.350 μmol m^−2^ s^−1^ and actinic light at 350 μmol m^−2^ s^−1^. The leaves from the control and treated plants were kept in the dark for 30 min to open the photosynthetic electron transport chain before analysis. For the chlorophyll *a* fluorescence parameters, we used the potential quantum yield of PSII—*F*_v_/*F*_m_ (Oxborough 2004), the light‐adapted quantum efficiency of PSII—ϕ_PSII_ (Genty et al. 1989), the nonphotochemical quenching in the steady state—NPQ (Horton and Ruban 1992), and the rate of fluorescence decline in steady‐state—*R*_fd_ (Lichtenthaler and Miehe 1997). Under water deficit conditions, reductions in *F*_v_/*F*_m_, ϕ_PSII_, and *R*_fd_ values may indicate a decline in photochemical efficiency in light and damage to the PSII reaction center (Maxwell and Johnson 2000). Meanwhile, the increase in NPQ values reflects the increase in the dissipation of excess light energy absorbed by PSII in the form of heat to protect the photosynthetic apparatus (Lichtenthaler et al. 2005; Demmig‐Adams et al. 2006, 2012).

### 2.4 Measurement of FWU

To assess foliar water uptake, one intact and mature leaf was collected from each *C. forbesii* and *C. guttata* individuals in both the control and treatment groups at 8 a.m. The leaf bases were immediately sealed with Vaseline paste for weighing. The leaves were then taken to the laboratory and weighed (fresh weight in g) using an analytical balance (Shimadzu AUY 220). After that, they were immersed in distilled water and weighed every 15 min for 2 h, every 30 min for 2 h, and then again after one more additional hour to obtain the foliar water uptake curve—FWU (final weight in g per unit of time; Liang et al. 2009). Before weighing each, the leaves were gently dried with absorbent microfiber towels to remove surface water. At the end of the experiment, leaf areas were measured using ImageJ software (AxionVision 1.54 g). The foliar water uptake curve (FWU) was calculated from the differences between fresh weight and final weight by leaf area using the equations: *FWU =* (fresh weight − final weight)/(leaf area). The rate of water capture by the leaves (WC) based on leaf area, was calculated using the equations: $\mathrm{WC}=\left(\text{Cmax}-\mathrm{Ci}\right)*\left(1-e^{-\mathrm{kt}}\right)$ + Ci, where *Cmax* is the maximum foliar water uptake, *Ci* is the initial foliar water content, *k* is the absorption exponential coefficient of the leaf (or the number of the absorption function curve), and *t* is the absorption time. Water capture by the leaves (WC) provides data considering the maximum foliar water uptake (C*max*; g cm^−2^ of leaf area) and the velocity of water absorption (*k*; Liang et al. 2009, modified; Pan et al. 2021).

### 2.5 Determination of the Diurnal Variation in Titratable Acidity

To evaluate changes in CAM metabolism expression in the leaves of both species, both in control plants and those subjected to drought after 30 days, we used the diurnal variation in titratable acidity (ΔH^+^). The diurnal variation in organic acids (ΔH^+^) was obtained from 200 mg of leaves collected at 6 a.m. and 6 p.m., and the difference in acidity accumulated during these two moments was represented by ΔH^+^. The samples were then weighed on a digital scale and boiled in 5 mL of distilled water for 5 min. The extracts (2.5 mL) were titrated with 0.01 M NaOH (pH 7.0) according to the protocol proposed by Hartsock and Nobel (1976). The results were expressed in μeq of H^+^ per g of fresh mass (FM).

### 2.6 Fluorescent Markers for Apoplastic Water Entry

To verify the apoplastic pathway of water entry into the leaves of *C. forbesii* and *C. guttata* , three mature leaves were collected from two individuals of each species in each treatment (*n* = 3). In the laboratory, the leaf bases were sealed with Vaseline paste, and 100 μL of a 1% aqueous solution of Lucifer Yellow (LY; lithium salt, Sigma) was applied to the adaxial and abaxial sides of each leaf using a Pasteur pipette. As a control, distilled water was applied to both surfaces of a third leaf (Supporting Information: 1). The solutions were applied to the middle third of the leaf, as described by Oparka and Read (1994). After LY was applied, the leaves were placed in gearbox boxes lined with filter paper moistened with distilled water. The boxes were then wrapped in aluminum foil, creating a dark chamber in which they remained for 5 h (Widholzer 2005; Eller et al. 2016). The excess marker on the leaves was subsequently washed off with distilled water, and freehand sections were obtained from the material where the marker had been applied. The slides were mounted in glycerin 50%, observed, and photomicrographed under an optical microscope with a fluorescence system (Leica DM4000 B LED, Leica) coupled to a camera (Leica DFC 3000 G), using the LAS X 1.1.0.12420 analysis software. Samples were excited with intense blue light (450–490 nm, FITC filter) with a 515 nm barrier filter (Oparka and Read 1994, modified).

### 2.7 Data Analysis

Mixed linear models (LMMs) were constructed to verify the difference in the potential quantum yield of PSII (F_v_/F_m_) of *C. forbesii* and *C. guttata* leaves between the analyzed times (0—control, 7, 14, 21, and 30 days), considering time (temporal replication) and species as fixed factors and individuals as random effects (using the *lmerTest* package: the *lmer* function; Kuznetsova et al. 2017). To determine whether there was a difference in the leaf structural parameters (LMA and SWC), leaf water balance (RWC), nonphotochemical quenching (NPQ), and rate of fluorescence decline in the steady state (*R*_fd_) between the control and treatment (water stress) and between each species ( *C. forbesii* and *C. guttata* ), as well as the interaction between treatments and species, a factorial ANOVA for independent samples was performed. These parameters met the assumptions of factorial ANOVA, presenting normality and homogeneity of variance. Considering the positive values of *F*_v_/*F*_m_ and ϕ_PSII_ (nonparametric data), Generalized linear models (GLMs) with a Gamma error family and inverse link function were constructed to verify differences between treatments and species (Akaike Information Criterion—AIC: *F*_v_/*F*_m_ = −57.04; ϕ_PSII_ = −48.64). The diurnal variation in organic acids between species and treatments was verified using GLM adjusting a ziGamma distribution (using the glmmTMB package; AIC: 76.70), given the presence of zero values and positive values (Brooks et al. 2023). To verify the variations in foliar water uptake (C*max* and *k*) between the control and treatment of the species, GLMs were constructed with a quasi‐Poisson distribution.

The GLMs were selected after verifying the homogeneity and normality of the model residues via the *DHARMa* package (Hartig 2022). To simplify the models, the interaction between species and treatments was removed from the parameter models (LMA, SWC, F_v_/F_m_, NPQ, C*max*, and *k*) when there was no significant effect. The means were compared using Tukey‐adjusted paired contrasts between treatments and species using the R package “emmeans” (Lenth 2023). For LMM, we calculated the marginal and conditional pseudo‐*R* ^2^ (Nakagawa et al. 2017) using the r.squaredGLMM function from the MuMIn package. The analyses were performed using R software (i386 4.5.0; R Core Team 2022), and the significance level was set at 5%.

To identify patterns of influence on the physiological parameters and leaf characteristics of *C. forbesii* and *C. guttata* in each treatment (control and drought), we performed a principal component analysis (PCA) using R software and the *factoextra* package (Kassambara and Mundt 2020). In PCA, all structural and physiological parameters were used as response variables, whereas species and treatments were considered complementary variables. The variables used in PCA were centered and scaled due to the different units.

## 3 Results

**FIGURE 2:**
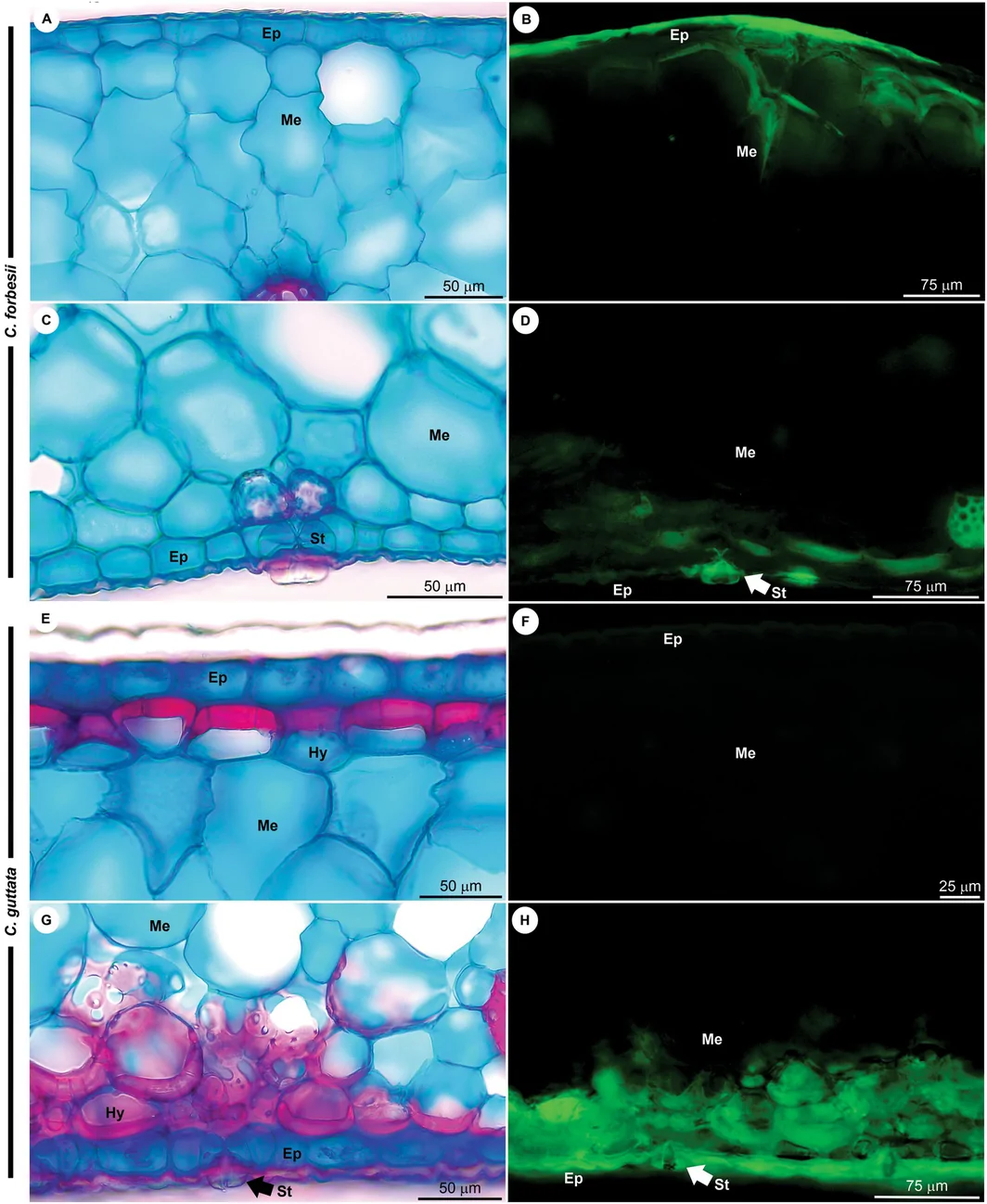
Leaf anatomy and water uptake assessed using Lucifer Yellow to trace the apoplastic pathway in *C. guttata* and *C. forbesii* . Internal structure (A, C) and the apoplastic pathway visualized with the fluorescent marker (B, D) in *C. forbesii* , and the same sequence in *C. guttata* (E–H). Abbreviations: Ep: Epidermis; Hy: Hypodermis; Me: Mesophyll; and St: Stomata (arrows).

The leaves of both *Cattleya* species were hypostomatic (Figure 2). In *C. forbesii* , the leaves were covered by a thick cuticle and had a uniseriate epidermis, composed of thin‐walled cells on the adaxial and abaxial surfaces (Figure 2A,C). The stomata were internally followed by two cells exhibiting reticulate wall thickenings. The mesophyll was homogeneous, consisting of large cells with small intercellular spaces (Figure 2A,C). In *C. guttata* , the adaxial surface was covered by a thick cuticle, and both surfaces exhibited a uniseriate epidermis. On the adaxial surface, the epidermis was followed by a hypodermis with thickened outer periclinal walls extending to the anticlinal walls (Figure 2E,G). Near the abaxial surface, the mesophyll comprised two to three cell layers with conspicuous wall thickenings (Figure 2G), remaining mesophyll cells were large and arranged with small intercellular spaces (Figure 2E,G).

Individuals of *C. forbesii* and *C. guttata* maintained under control conditions and subjected to drought for 30 days presented distinct responses in terms of leaf water content, foliar water uptake rate, and chlorophyll *a* fluorescence.

### 3.1 Variations in Leaf Structure and Water Balance

**FIGURE 3:**
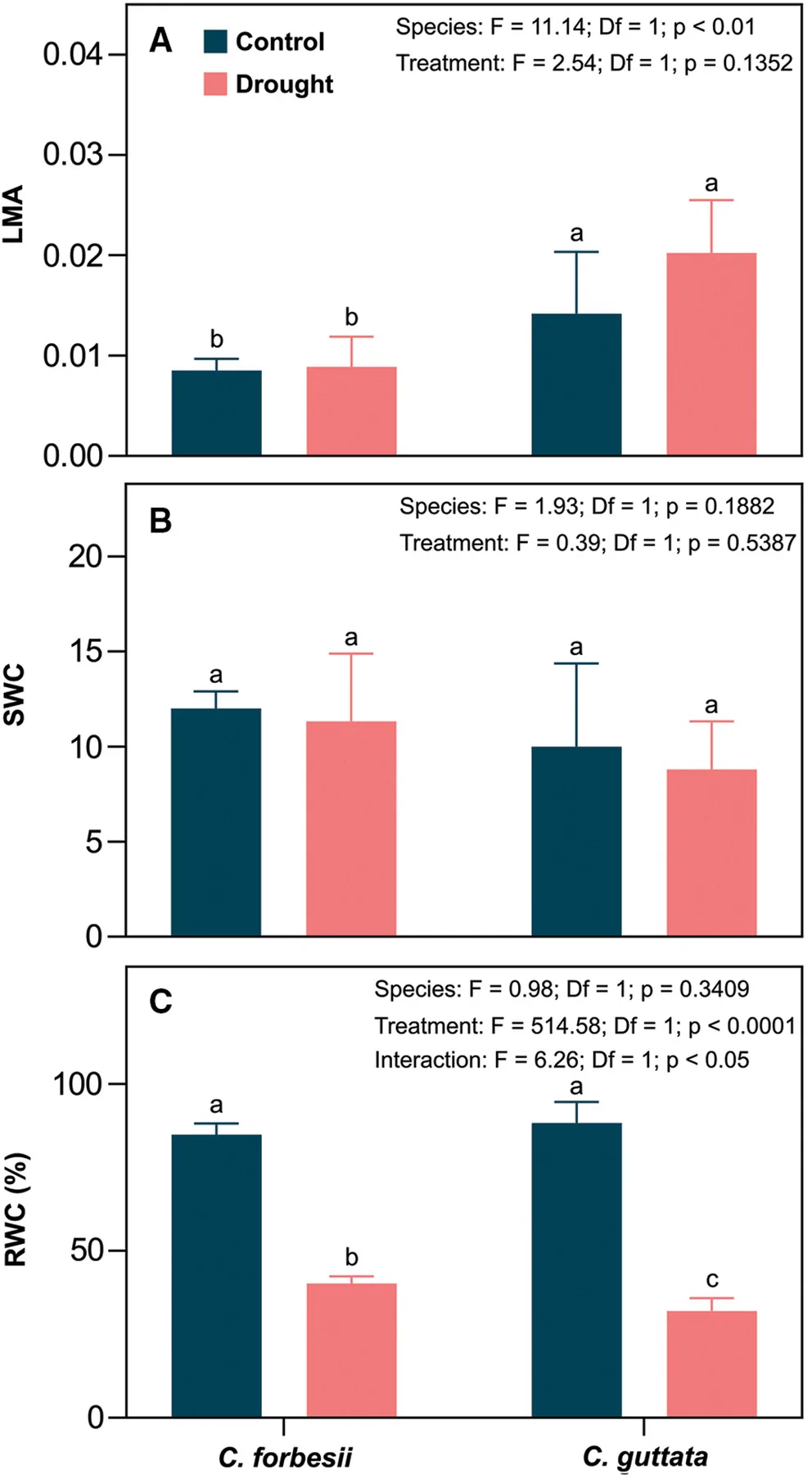
Variations in the leaf mass per area and leaf water balance of individuals of *C. forbesii* and *C. guttata* under control conditions and those subjected to drought for 30 days. (A) LMA—leaf mass per area; (B) SWC—saturated water content; and (C) RWC—relative water content. Lowercase letters represent significant differences between predictor variables, without interaction between them, using Factorial Analysis of Variance (*p* < 0.05), and we compare the means using Tukey's adjusted paired comparisons (“emmeans” package; *p* < 0.05).

No significant changes were observed in leaf mass per area (LMA) or water saturation content (SWC) when the plants were subjected to drought (Figure 3A,B). In general, *C. guttata* has leaves with significantly higher leaf mass (LMA) compared to *C. forbesii* (Figure 3A). However, there was no difference in SWC between the species (Figure 3B). There was a significant difference in relative water content (RWC) in relation to species and treatment (control and drought; Figure 3C). After being subjected to water stress, the plants in the treatment presented a reduction of approximately 44% in the relative water content (RWC) in *C. forbesii* and 56% in *C. guttata* (Figure 3C).

### 3.2 Chlorophyll a Fluorescence

**FIGURE 4:**
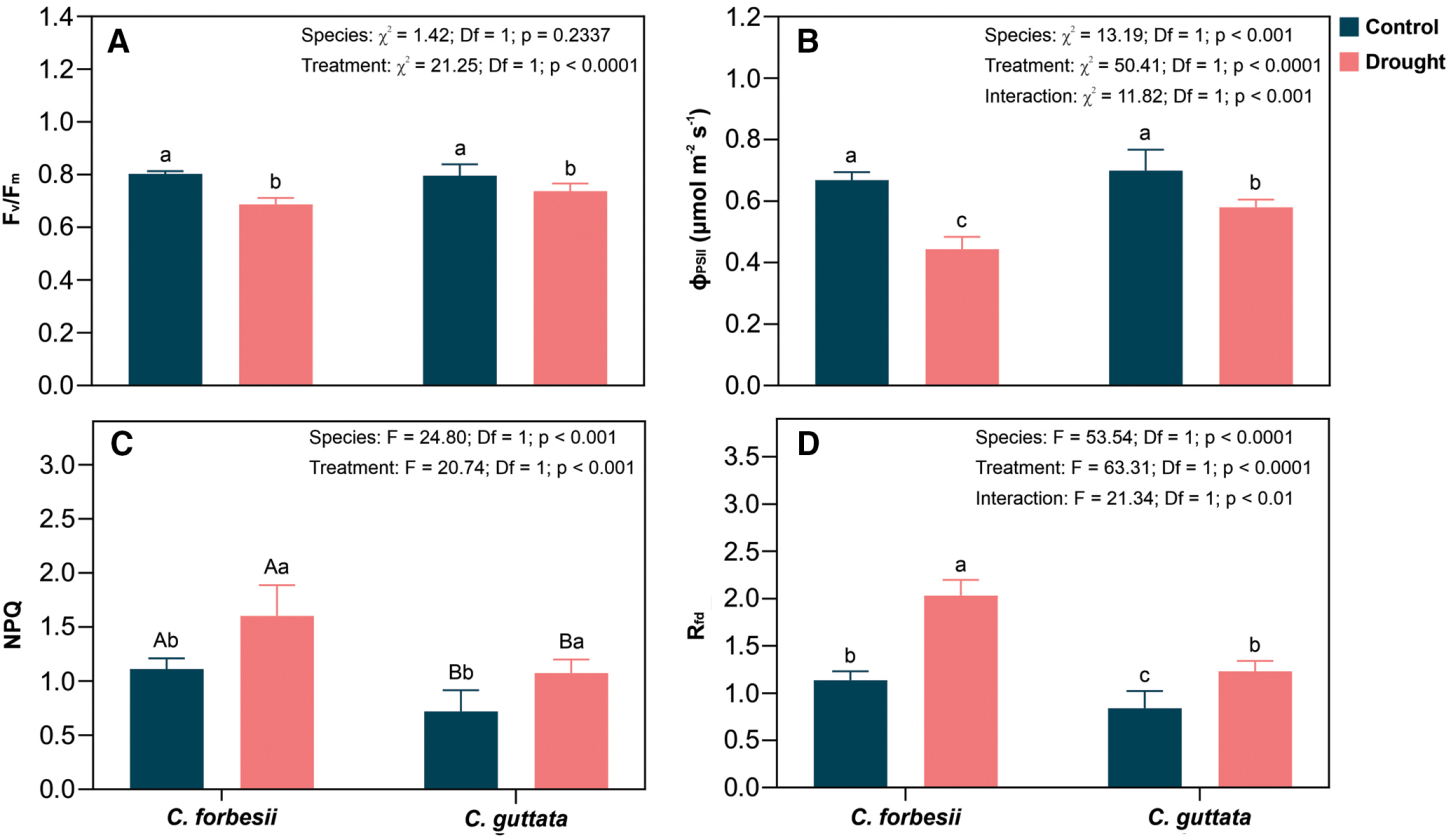
Chlorophyll *a* fluorescence was measured in individuals of *C. forbesii* and *C. guttata* under control conditions and those subjected to drought for 30 days. (A) Potential quantum yield of PSII (*F*_v_/*F*_m_); (B) light‐adapted quantum yield of PSII (ϕ_PSII_). (C) Nonphotochemical quenching (NPQ); (D) rate of fluorescence decline in steady‐state (*R*_fd_). Individuals in the control group are represented by navy blue bars, and individuals subjected to water stress are represented by pink bars. Changes in letters indicate significant differences between predictor variables (species, uppercase letters; treatments, lowercase letters) when the GLM with gamma distribution (*F*_v_/*F*_m_ and ϕ_PSII_) and factorial ANOVA (NPQ and *R*_fd_; *p* < 0.05) were used, and in both cases, the means were compared using Tukey's adjusted paired comparisons (“emmeans” package; *p* < 0.05).

The highest *F*_v_/*F*_m_ and ϕ_PSII_ values were observed in individuals from the control group of both species, with a reduction when subjected to drought for 30 days (Figure 4A,B). Compared with *C. guttata* , *C. forbesii* presented greater decreases in ϕ_PSII_ values. In general, NPQ values were greater in *C. forbesii* . In both species, drought promoted an increase in NPQ values (Figure 4C). The highest *R*_fd_ values were detected in *C. forbesii* individuals in the treatment group, and the lowest *R*_fd_ values were detected in *C. guttata* control individuals (Figure 4D). In both species, the induction of drought significantly increased the *R*_fd_ values.

### 3.3 Diurnal Variation in Titratable Acidity

Compared with *C. guttata* , which had an average of 6.16 μeq H^+^ g^−1^ FM, *C. forbesii* individuals showed a significantly greater diurnal variation in organic acids (ΔH^+^), with an average of 24.42 μeq H^+^ g^−1^ FM (Species: *χ* ^2^ = 12.72; Df = 1; *p* < 0.001). However, neither species showed a significant difference in diurnal variation in leaf acidity between individuals in the control group and those subjected to drought for 30 days (Treatment: *χ* ^*2*^ = 1.37; Df = 1; *p* = 0.2410).

### 3.4 Foliar Water Uptake and Fluorescent Apoplastic Markers

**FIGURE 5:**
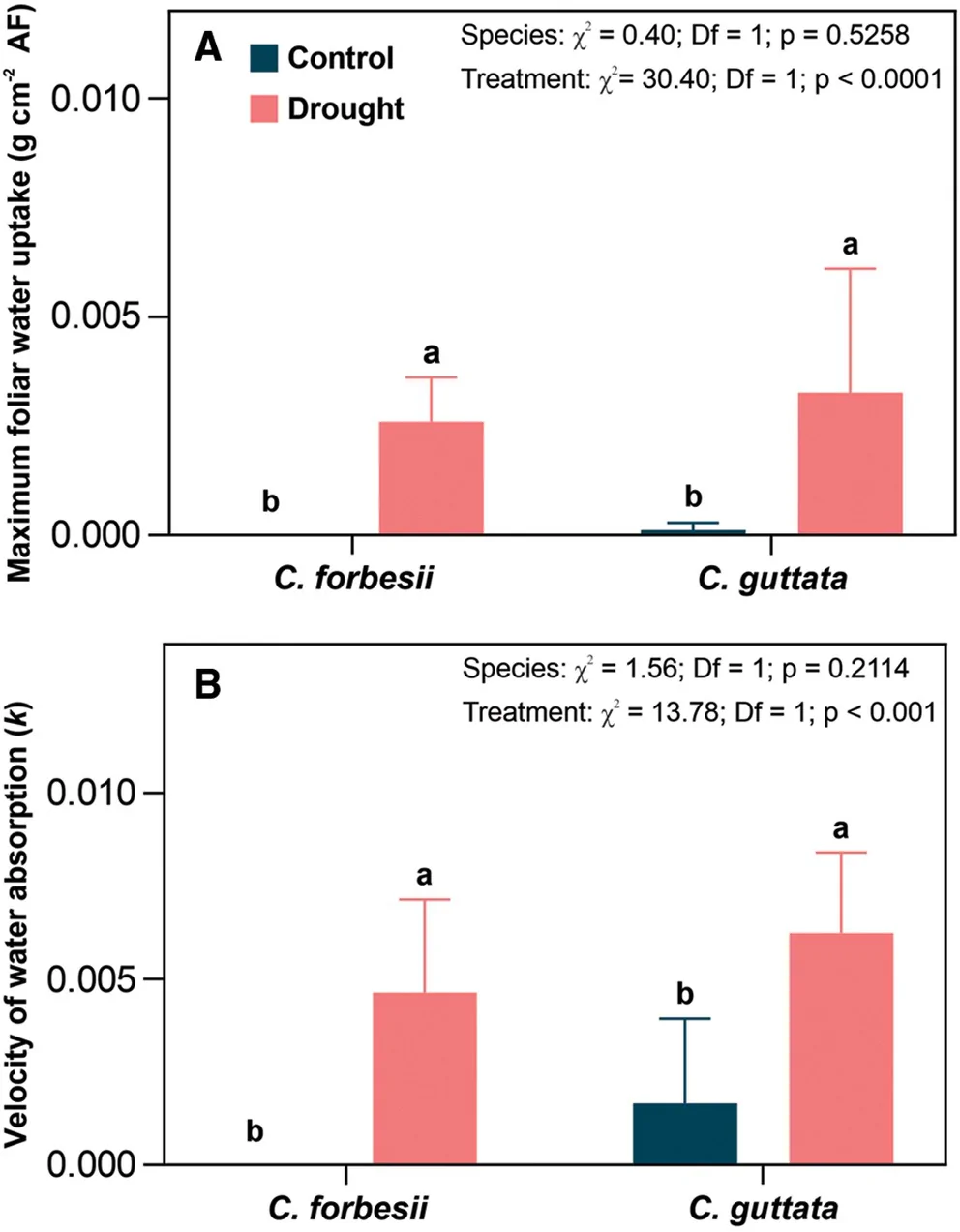
Variations in the parameters of foliar water uptake of hydrated individuals of *C. forbesii* and *C. guttata* and those that experienced 30 days of drought. (A) The bar graphs represent changes in the maximum foliar water uptake (C*max*) and (B) velocity of water absorption (*k*) in the control and treatment groups of *C. forbesii* and *C. guttata* . Different letters represent significant differences between species (capital letters) and treatments (lowercase letters), as determined by GLM with a quasi‐Poisson distribution, and we compared the means using Tukey's adjusted paired comparisons (“emmeans” package; *p* < 0.05).

Significantly higher rates of maximum foliar water uptake (higher C*max* values) and velocity of water absorption (higher *k* values) were observed in individuals of *C. guttata* and *C. forbesii* after 30 days of drought (Figure 5A,B). The apoplastic fluorescent marker Lucifer Yellow stained the cuticle, epidermal cell walls, and some mesophyll cells of the adaxial and abaxial surfaces of *C. forbesii* leaves (Figure 2B,D), whereas in *C. guttata*, LY was detected only on the abaxial surface (Figure 2F,H).

**FIGURE 6:**
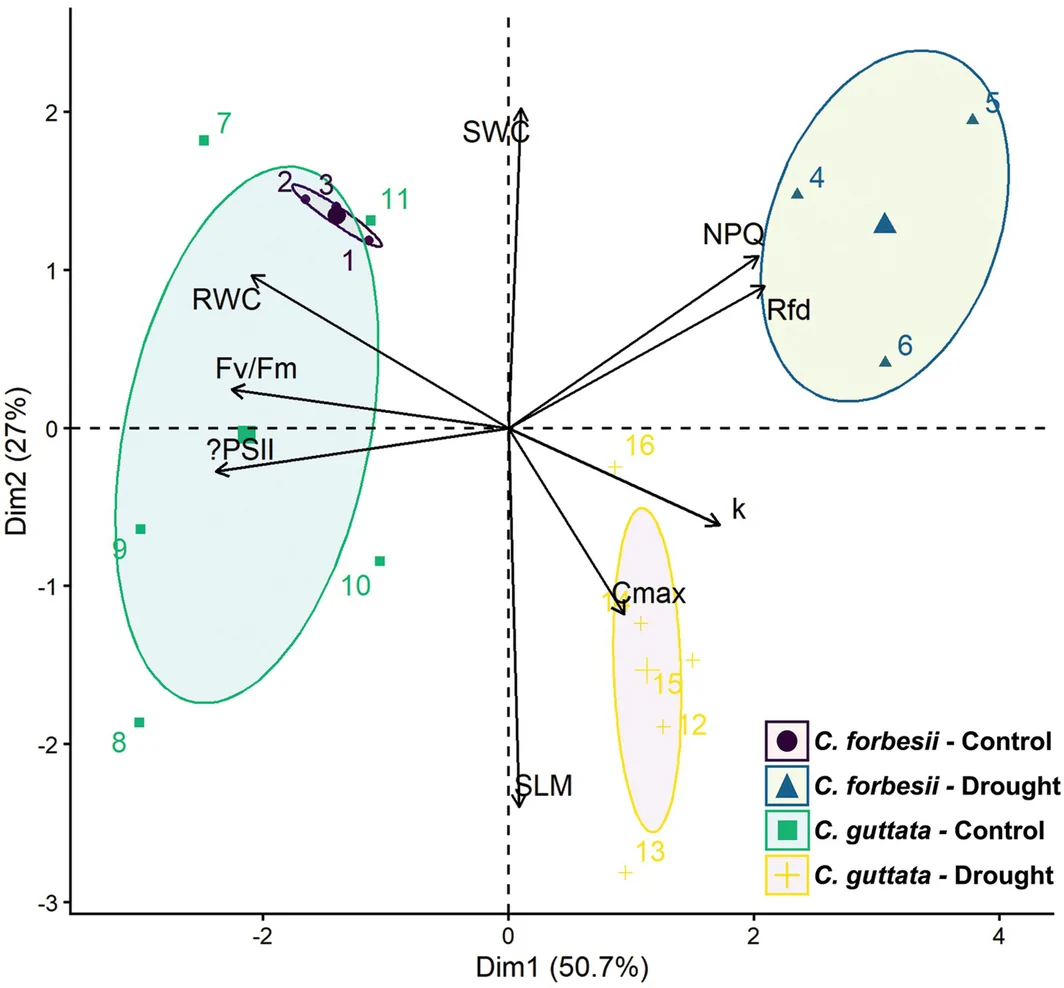
Principal component analysis (PCA) was performed to verify the relationships among chlorophyll *a* fluorescence, leaf water status, structural characteristics, and foliar water uptake, as well as between *C. forbesii* and *C. guttata* and between the control and the drought treatment. The variables included LMA (leaf mass per area), SWC (saturated water content), RWC (relative water content in leaves), *F*_v_/*F*_m_ (potential quantum yield of PSII), ϕ_PSII_ (light‐adapted quantum yield of PSII), NPQ (nonphotochemical quenching), *R*_fd_ (the rate of fluorescence decline in steady‐state), C*max* (maximum foliar water uptake), and *k* (velocity of water absorption).

Principal component analysis (PCA) revealed that the first two components accounted for approximately 77.5% of the total variance in the data, indicating that the variables included contributed strongly to discrimination between control plants and those subjected to 30 days of drought (Figure 6). We observed that the control individuals of *C. forbesii* and *C. guttata* were differentiated from those subjected to drought (Dim. 1%–50.7% explanation), which is related to the RWC, photochemical parameters (F_v_/F_m_, ϕ_PSII_, NPQ, and *R*_fd_), and FWU values (C*max* and *k*). In Dim. 2 (27%), *C. forbesii* and *C. guttata* individuals subjected to drought for 30 days were separated. *C. forbesii* was influenced by NPQ and *R*_fd_ values, whereas *C. guttata* was influenced by LMA values and parameters related to the FWU.

## 4 Discussion

The induction of drought altered the leaf water status, photosynthetic activity, and water absorption capacity of the leaves of both *Cattleya* species. After 30 days of drought, the leaf water status and photosynthetic activity of both species decreased (with lower RWC, *F*_v_/*F*_m_, and ϕ_PSII_ values), but nonphotochemical quenching (NPQ) and index values indicating photosystem vitality (*R*_fd_) increased. Despite low C*max* values and no variation in organic acid accumulation, the rate of foliar water uptake increased after drought, partially corroborating our hypothesis. Regarding the species studied, we observed that *C. guttata* showed higher LMA compared to *C. forbesii* . Non‐photochemical quenching (NPQ), one of the most important photoprotective mechanisms, was greater in *C. forbesii* compared to *C. guttata* , indicating an investment in increased dissipation of excess light energy. FWU may be a strategy for the *Cattleya* species studied during periods of water deficit. Both species showed a significant reduction in RWC at the end of the experiment, which may have consequently boosted their FWU capacity.

### 4.1 Effect of Water Stress on the Leaf Water Balance

As an alternative to constant fluctuations in water availability, several epiphytic species, including orchids, can store water in their tissues, a phenomenon known as leaf succulence (Oliveira and Sajo 1999; Pires et al. 2012; Moreira et al. 2013; Guevara Pérez et al. 2019). In our study, the leaf succulence of *Cattleya* species was not affected by water stress, maintaining SWC values even after 30 days of water restriction. High SWC values can be associated with a cellular and tissue‐level strategy to escape water‐deficient conditions (Ogburn and Edwards 2012). This is because cellular activities are maintained by the presence of water in leaf cells, even under environmental conditions of water restriction (Ogburn and Edwards 2012). The structural characteristics associated with leaf succulence include a mesophyll composed of several cell layers, whose cells are large, with thin walls and vacuoles, and have few cell spaces (Ogburn and Edwards 2012; Zhang et al. 2018; Lamont and Lamont 2025). In addition, some cell wall arrangements may increase wall plasticity and favor succulence (Fradera‐Soler et al. 2022). In particular, the elasticity of succulent plant walls is associated with a high degree of methyl esterification of homogalacturonans (HGs) and a high abundance of type I rhamnogalacturonans (RG‐I), which confer dynamic properties to the cell wall. The degree of leaf succulence varies between species and depends on the relative composition and arrangement of dry tissue mass, water, and air (Lamont and Lamont 2025). In general, tropical orchid leaves have a high‐water content (Perez‐Lopez et al. 2023) and may be classified as semi‐succulent (or semi‐succophyll, according to Lamont and Lamont 2025). Because epiphytic plants depend on the atmosphere as a source of water and nutrients, they may be subject to severe variations in water availability at certain times of the year or even during the day (Zotz and Bader 2009; Zhang et al. 2016). Succulence can reduce the rate of water deficit in tissues during the day and protect against daily fluctuations and seasonal variations in water loss (Perez‐Lopez et al. 2023). Even with leaves formed by tissues that favor water storage, some epiphytic species show a reduction in the relative water content and water potential of their leaves during periods of water deficit (de la Rosa‐Manzano et al. 2017; Chávez‐Sahagún et al. 2019; Tay et al. 2015, 2019), as we observed in both *Cattleya* species used in this study. In other words, water restriction impacts the amount of water present in leaf tissues of the species (reduction in RWC), compromising basic metabolic processes (*F*_v_/*F*_m_ and ɸ_PSII_).

Lamont and Lamont (2025) reported that leaf area per area (SLA—area per unit mass) is not directly related to leaf succulence but can serve as an index of drought tolerance because of its relationship with leaf thickness, that is, with the volume and number of cells present in the mesophyll. LMA values, in turn, are inversely related to SLA values and are generally greater in epiphytic orchids than in terrestrial orchids, as is the case for water saturation content, which corroborates a positive relationship between these attributes, as proposed by Zhang et al. (2015, 2018). Despite similar SWC values among the species studied, the highest LMA values were observed in the leaves of *C. guttata* . We speculate that, in this species, water storage occurs in a larger number of cells rather than in larger cells.

### 4.2 Effect of Water Stress on Photochemical Efficiency

Low water availability in the environment can affect the water availability of leaves (reducing their relative water content) of epiphytic orchids and, consequently, their photosynthetic efficiency (Tay et al. 2015). Despite the low accumulation of organic acids during the night observed in our work, the *C. guttata* and *C. forbesii* individuals used in this study were the same as those used by Lima et al. (2023), presented carbon isotopic signature values (δ^13^) of 16.49‰ and −14.95‰, respectively, and were classified as CAM. Many orchids use the CAM pathway as a strategy to cope with fluctuations in water availability in the epiphytic environment (Cushman 2001; Silvera et al. 2009; Moreira et al. 2013; Kerbauy et al. 2012; Zhang et al. 2018), with the intensity of the CAM pathway expression varying between different plant organs or even depending on water availability (Rodrigues et al. 2013). Although water restriction for 30 days affected the water status (RWC) in the leaves of *C. forbesii* and *C. guttata* , it was not able to intensify the expression of CAM metabolism (diurnal variation in organic acids—ΔH^+^).

Although no changes were observed in the expression of CAM metabolism when the plants were subjected to drought, *Cattleya* leaves experienced a reduction in photosynthetic efficiency. A reduction in photochemical efficiency during a period of drought has previously been reported in other epiphytic orchids (Stancato et al. 2001; Lüttge 2010; Tay et al. 2015; de la Rosa‐Manzano et al. 2017), and chlorophyll *a* fluorescence has been established as a sensitive tool for diagnosing water stress, even in CAM plants. Our results suggest that the photosynthetic apparatus of both species was compromised after drought (lower *F*_v_/*F*_m_ and ϕ_PSII_ values). Monitoring chlorophyll fluorescence parameters in CAM plants under water stress conditions has revealed a reduction in the electron transport rate and ϕ_PSII_ (de Mattos et al. 1999; Zheng et al. 2019; Ceusters et al. 2019). For example, the *F* *_v_*/*F* *_m_* values of species such as *Kalanchoë daigremontiana* and *Opuntia ficus‐indica* decrease after dehydration (Borland et al. 2014), reaching levels correlated with the occurrence of photoinhibition (Maxwell and Johnson 2000).

To prevent damage to the photosynthetic apparatus, plants employ various photoprotective strategies, such as the thermal dissipation of light energy (Pieters et al. 2003; Demmig‐Adams et al. 2006; Sharma et al. 2020). Heat dissipation prevents the formation of reactive oxygen species (ROS) and is measured from the perspective of nonphotochemical quenching (NPQ) of PSII chlorophyll fluorescence (Niyogi 2000; Demmig‐Adams et al. 2006). Under stress conditions, chlorophyll de‐excitation through the xanthophyll cycle results in nonphotochemical energy dissipation, which increases NPQ (Adams and Demmig‐Adams 1996; Holt et al. 2005; Tay et al. 2019), as we observed in both species under water restriction (high NPQ values). In addition, dimension 1 of the PCA revealed that *C. forbesii* and *C. guttata* individuals subjected to water stress were related to NPQ and the rate of fluorescence decline in steady‐state (R_fd_), indicating the relevance of these parameters under drought. Primarily for *C. forbesii* , as represented in dimension 2 of the PCA, we observed an influence of nonphotochemical extinction and rate of fluorescence decline in the steady‐state (*R*_fd_) in individuals subjected to water restriction. Similarly, in other epiphytic orchids, the dissipation of excess energy absorbed by PSII increases because of seasonal drought, whereas the maximum quantum efficiency of PSII decreases (de la Rosa‐Manzano et al. 2015, 2017). As in these orchids, the nonphotochemical energy dissipation strategy may help *Cattleya* species cope with water stress during the dry season, functioning as an efficient photoprotective mechanism.

### 4.3 Effect of Water Stress on Foliar Water Uptake (FWU)

The induction of water stress compromised the water status of both *Cattleya* species (low RWC values), possibly contributing to an increase in the FWU (higher C*max* values). However, the variation in organic acid accumulation (ΔH^+^) apparently does not influence FWU capacity. Foliar water uptake phenomenon occurs when atmospheric water exceeds the leaf surface in response to a water potential gradient, promoting tissue hydration (Burgess and Dawson 2004; Dawson and Goldsmith 2018; Steppe et al. 2018). In epiphytes, the ability to absorb water through leaves is an alternative uptake strategy that helps species cope with water scarcity (Gotsch et al. 2015; Pan et al. 2021; Lima et al. 2023). When atmospheric humidity increases, such as in the early morning, FWU helps maintain water status and can improve stomatal conductance and photosynthetic activity (Limm et al. 2009; Eller et al. 2013; Berry et al. 2014, 2019; Guzmán‐Delgado et al. 2018).

In a climate change scenario, which predicts an increase in drought events and a reduction in the leaf water potential (the driving force of FWU; Eller et al. 2016; Dawson and Goldsmith 2018; Schreel et al. 2020), FWU may represent an alternative pathway for water acquisition and to alleviate water stress in *C. forbesii* and *C. guttata* . These species occur in the Atlantic Forest and, particularly in montane forests (1000 m above sea level), fog events are frequent (Rosado et al. 2010). Although there was no difference in foliar water uptake (FWU) between species, considering individuals subjected to drought, dimension 2 of the PCA indicated a relationship between C*max* and *k* in individuals of *C. guttata* . The Ministry of the Environment (Ordinance No. 443, dated December 17, 2004) includes *C. guttata* on the list of endangered species. Our results indicate that, under water‐restricted conditions, the leaf water absorption capacity of this species may help it cope with the constant fluctuations in water availability in the epiphytic environment and its adaptation to environmental changes. However, future experiments are needed to effectively test the effect of FWU on plant performance and survival during drought, as well as the contribution of exposure to events that promote leaf hydration, such as fog.

Several leaf traits may favor FWU, including more negative leaf water potentials, low dry matter content, elastic cell walls, and high stomatal and cuticular conductance (Matos et al. 2024). Foliar water uptake can occur through stomata, trichomes, hydathodes, or the cuticle (Eller et al. 2016; Fernández et al. 2017; Losada et al. 2021; Gui et al. 2021; Guzmán‐Delgado et al. 2021; Fradera‐Soler et al. 2024). Because cuticular permeability depends on its structure and chemical composition, differences between leaf surfaces may influence FWU patterns. Cuticle permeability is generally affected by lipids, such as waxes and cutin (hydrophobic compounds), as well as polysaccharide (hydrophilic compounds) compositions (Fernández et al. 2016, 2017; Guzmán‐Delgado et al. 2016). In our study, the apoplastic fluorescent marker LY indicated that both *Cattleya* species were capable of absorbing water through the leaf surface, although distinct patterns were observed between species. *C. forbesii* absorbed water through both the adaxial and abaxial surfaces, whereas *C. guttata* absorbed water only through the abaxial surface, which may indicate differences in cuticle composition and permeability, since the marker was not detected on the adaxial surface. Despite the presence of a hypodermis with highly lignified outer periclinal walls in *C. guttata* , this structure does not appear to function as a barrier to water movement. In contrast, the stronger fluorescence observed in mesophyll cells near the abaxial surface may indicate that their lignified cell walls contribute to the apoplastic water flow within the leaf tissues.

## 5 Conclusions

In this study, we observed how water restriction can influence water status, photosynthetic performance, and leaf water absorption capacity in two epiphytic orchids: *C. forbesii* and *C. guttata*. In hydrated plants, the relative water content in the leaves and photosynthetic activity remained high, but the FWU rate was low. After water restriction, both *Cattleya* species showed a reduction in leaf water status accompanied by an increase in FWU (C*max* values). Despite this increase in FWU, photosynthetic activity was still negatively affected. Our results indicate that the greater amount of water in the leaf tissue (semi‐succulent leaves) of both species may limit the FWU, whereas water scarcity (dry period) may increase this capacity. The apoplastic fluorescent marker Lucifer Yellow (LY) was predominantly detected in the cuticle of both species; however, water uptake occurred through both leaf surfaces in *C. forbesii* , whereas in *C. guttata* absorption was restricted to the abaxial surface. Although our results indicate that FWU can occur in both species, experimental approaches combining drought and leaf‐wetting events are necessary to determine whether this pathway effectively mitigates water stress and enhances plant performance under natural conditions.

## Author Contributions

Jéssica Ferreira de Lima contributed to experimental design, data collection, data analysis, statistical processing, and manuscript writing. Gisely Souza dos Santos contributed to the data collection, reviewing, and editing of the manuscript. Denis Coelho de Oliveira assisted in data collection and analysis, as well as in the review and editing of the manuscript. Ana Silvia Franco Pinheiro Moreira, as supervising author, contributed to the development of the concept and the review and editing of the manuscript.

## Funding

This work was supported by Coordenação de Aperfeiçoamento de Pessoal de Nível Superior (CAPES, JFL), Conselho Nacional de Desenvolvimento Científico e Tecnológico (CNPQ, ASFPM), Fundação de Amparo à Pesquisa do Estado de Minas Gerais (FAPEMIG), and Programa de Pós‐graduação em Ecologia, Conservação e Biodiversidade (PPGECO) of Universidade Federal de Uberlândia (UFU).

## Conflicts of Interest

The authors declare no conflicts of interest.

## Supporting information


**Supporting Information 1:** Control leaves, with distilled water applied to both surfaces, for analysis of the apoplastic pathway visualized with the fluorescent marker (Lucifer Yellow) in *C. forbesii* (A), and in *C. guttata* (B). *Acronyms*: Ep: epidermis and Me: mesophyll.

## Data Availability

The data that support the findings of this study are available on request from the corresponding author. The data are not publicly available due to privacy or ethical restrictions.
