# Supplementary material for: Changes in Foliar Water Uptake Caused by Water Stress in Cattleya Lindl. (Orchidaceae)
Source: Physiol Plant. 2026 Sep 2;178(5):e71047. doi: 10.1111/ppl.71047 (PMC13536186; doi:10.1111/ppl.71047)
Supplement: Supplementary file 1 [file PPL-178-e71047-s001.docx]

**Changes in foliar water uptake caused by water stress in *Cattleya* Lindl. (Orchidaceae)**

Jéssica Ferreira de Lima^1^, Gisely dos Souza Santos^1^, Denis Coelho de Oliveira^2^ and Ana Silvia Franco Pinheiro Moreira^1,*^

^1^Instituto de Biologia, Laboratório de Fisiologia Vegetal, Universidade Federal de Uberlândia (UFU), Uberlândia, Minas Gerais, Brazil

^2^Instituto de Biologia, Laboratório de Anatomia, Desenvolvimento Vegetal e Interações, Universidade Federal de Uberlândia (UFU), Uberlândia, Minas Gerais, Brazil

^*^Corresponding author: [anasilviamoreira@gmail.com](mailto:anasilviamoreira@gmail.com)

Suppl. 1: Control leaves, with distilled water applied to both surfaces, for analysis of the apoplastic pathway visualized with the fluorescent marker (Lucifer Yellow) in *C. forbesii* (A), and in *C. guttata* (B). *Acronyms*: Ep: epidermis and Me: mesophyll.
